# Role of Decompressive Surgery in Neurologically Intact Patients with Low to Intermediate Intraspinal Metastatic Tumor Burden

**DOI:** 10.3390/cancers15020385

**Published:** 2023-01-06

**Authors:** Niklas von Spreckelsen, Julian Ossmann, Maximilian Lenz, Lukas Nadjiri, Moritz Lenschow, Sergej Telentschak, Johanna Meyer, Julia Keßling, Peter Knöll, Peer Eysel, Roland Goldbrunner, Moritz Perrech, Max Scheyerer, Eren Celik, Kourosh Zarghooni, Volker Neuschmelting

**Affiliations:** 1 Department of General Neurosurgery, Center for Neurosurgery, Cologne University Hospital, Faculty of Medicine and University Hospital, University of Cologne, 50937 Cologne, Germany; 2 Department of Orthopedics and Trauma Surgery, University of Cologne, 50937 Cologne, Germany; 3 Department of Radiooncology and Cyberknife Center, University of Cologne, 50937 Cologne, Germany

**Keywords:** ESCC, MSCC, spinal metastasis, decompressive surgery, neurological outcome

## Abstract

**Simple Summary:**

Metastatic spinal disease with epidural spinal cord compression (ESCC) is a devastating disease. Treatment regimens are interdisciplinary and often combine surgery, radiotherapy, and medical treatment. For neurologically intact patients with ESCC, there are limited data on whether decompressive surgery is necessary to preserve neurological function.

**Abstract:**

Background: Surgical decompression (SD) followed by radiotherapy (RT) is superior to RT alone in patients with metastatic spinal disease with epidural spinal cord compression (ESCC) and neurological deficit. For patients without neurological deficit and low- to intermediate-grade intraspinal tumor burden, data on whether SD is beneficial are scarce. This study aims to investigate the neurological outcome of patients without neurological deficit, with a low- to intermediate-ESCC, who were treated with or without SD. Methods: This single-center, multidepartment retrospective analysis includes patients treated for spinal epidural metastases from 2011 to 2021. Neurological status was assessed by Frankel grade, and intraspinal tumor burden was categorized according to the ESCC scale. Spinal instrumentation surgery was only considered as SD if targeted decompression was performed. Results: ESCC scale was determined in 519 patients. Of these, 190 (36.6%) presented with no neurological deficit and a low- to intermediate-grade ESCC (1b, 1c, or 2). Of these, 147 (77.4% were treated with decompression and 43 (22.65%) without. At last follow-up, there was no difference in neurological outcome between the two groups. Conclusions: Indication for decompressive surgery in neurologically intact patients with low-grade ESCC needs to be set cautiously. So far, it is unclear which patients benefit from additional decompressive surgery, warranting further prospective, randomized trials for this significant cohort of patients.

## 1. Introduction

Spinal metastatic disease (MSD) with malignant epidural spinal cord compression (ESCC) is a devastating illness with a stark impact on individual independence in everyday life and overall prognosis [1,2,3,4]. Likely due to advances in the treatment of primary cancers and the corresponding prolonged survival of patients, as well as more readily available imaging, the incidence of MSD and ESCC has been increasing over the past years [5,6]. In a landmark study of patients with a neurological deficit due to ESCC, Patchell et al. showed that decompressive surgery prior to radiotherapy led to a better neurological outcome when compared to radiotherapy alone [7]. Therefore, surgical decompression is routinely performed as part of the multimodal treatment for patients with ESCC and neurologic deficits [8,9,10]. In neurologically intact patients, treatment indications are often set accordingly and reasoned with “impending” paralysis due to intraspinal metastatic growth in order to preserve the neurological outcome. Since Patchell et al. reported only on patients with neurological deficits, the evidence supporting decompressive surgery in this patient cohort with low-grade ESCC and preserved neurological function is far less clear [7]. This work, therefore, aimed to investigate the role of decompressive surgery on the neurological outcome of patients with MSD presenting with mild to intermediate ESCC and no neurological deficits

## 2. Materials and Methods

### 2.1. Patient Selection

In a retrospective analysis, patients treated between March 2011 and March 2021 for spinal metastases at our institution were screened. Clinical, pathologic, and imaging data were collected and analyzed after approval by the local ethics committee. (Approval code: 20-1643.)

### 2.2. Clinical Analysis

The analyzed parameters included demographic parameters such as age and gender, general clinical characteristics such as Karnofsky Performance Status (KPS), primary tumor origin (multiple myeloma and lymphoma were summarized as hematopoietic cancers), medical comorbidities were defined as a history of smoking, obesity (defined as a body mass index > 30), coronary heart disease, chronic obstructive pulmonary disease (COPD), diabetes mellitus, history of deep vein thrombosis or pulmonary embolism (PE), osteoporosis and current glucocorticoid therapy at the time of treatment. Treatment-related complications included wound infection, thrombosis/PE, pneumonia, other, and delay of adjuvant treatment due to complications.

The neurological status was recorded at first presentation as well as at last follow-up and classified according to the modified Frankel score (A = complete impairment; B = incomplete, sensory but no motor function below neurological level; C = incomplete, motor function preserved but majority of key muscles muscle grade < 3; D = incomplete, motor function preserved and majority of key muscles muscle grade > 3; E = normal) [11,12]. Patients presenting with a Frankel score of D and E were deemed ambulatory. Changes in pre- and postoperative Frankel scores were used to assess neurological outcomes and defined as improvement (increase in at least one Frankel grade), stable (no change in Frankel grade), or worsening (decrease in at least one grade).

As an objective parameter for the grade of intraspinal tumor burden, the ESCC scale was determined for each patient based on preoperative CT or MRI imaging data of the corresponding spine [13]. For patients to be included, CT scans with axial and sagittal slices of at least 5 mm thickness with bone and soft tissue windows and/or magnetic resonance imaging (MRI) with contrast-enhanced and non-contrast T1 as well as T2 sequences were required. Spinal stability was assessed using the Spinal Instability Neoplastic Score (SINS) using location, pain, bone lesion, radiographic spinal alignment, vertebral body collapse, and posterior spinal element involvement [14,15]. Patients were categorized into a ‘stable’ (SINS 0–6), ‘intermediate’ (SINS 7–12), or ‘instable’ (SINS 13–18) group.

**Figure 1:**
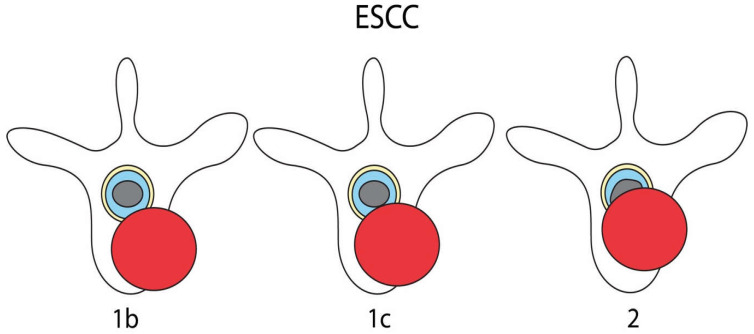
Illustration of included ESCC grades adopted from Bilsky et al. [13]. (1b) Deformation of the thecal sac, without spinal cord abutment; (1c) Deformation of the thecal sac, with spinal cord abutment, without cord compression; (2) Spinal cord compression, with cerebrospinal fluid (CSF) visible around the cord.

All patients who initially presented with an ESCC scale of 1b, 1c, or 2 (Figure 1) and a Frankel score of E were included in this study. In the case of any neurological deficit (Frankel A–D), missing or incomplete imaging or clinical data records, and in the case of omitted treatment (e.g., palliative and best supportive care), patients were excluded. In cases with multiple lesions, the surgically treated level or level with the highest grade of ESCC was reported for the case. If patients presented with a new lesion at a different spinal level 3 months or more after initial treatment, those cases were analyzed as separate cases. Patients were dichotomized according to whether they were receiving decompressive surgery or not. Patients treated with instrumentation only without intraspinal decompression were categorized into the “no decompressive surgery” group accordingly.

The treatment strategies for each patient were determined by a tumor board panel or the treating surgeon/radiation oncologist. Treatment modalities included (1) decompressive surgery, (2) decompressive surgery and instrumentation, (3) instrumentation without decompression or vertebroplasty, and (4) no surgical intervention/RT only. Adjuvant radiotherapy was scheduled for all patients, and adjuvant medical systemic tumor therapy if applicable. There were no institutional protocols for surgical/non-surgical treatment; each treatment strategy catered to patient- and case-specific findings and the overall assessment by the treating board-certified surgeon and/or radiation oncologist.

### 2.3. Statistical Analysis

Statistical analysis was performed with SPSS software (Version 28, IBM SPSS Statistics, Armonk, NY, USA). A *p* < 0.05 was considered statistically significant. Categorical variables were compared using the chi-square or Fisher’s Exact test when applicable. Normal distribution in continuous variables was tested using the Kolmogorov–Smirnov test. A two-sided unpaired Student’s t-test was used to compare normally distributed group means, while a Mann–Whitney U test was used in cases of non-normal or heteroscedastic data distribution. Data are reported as mean ± standard deviation (SD) or median (95% confidence interval).

## 3. Results

The initial analysis included 519 cases with complete clinical and imaging data. Of these, 181 (34.9%) presented without neurological deficit (Frankel E) and a low or intermediate ESCC scale (1b-2) (Figure 1) and were selected for further analysis. Median follow-up was 3 months (SD 14.8; min. 0, max. 79 months).

### 3.1. Patient Characteristics

The patient cohort (*n* = 181) included 104 (57.5%) men and 77 (42.5%) women. Median age at presentation was 65 years (min. 13, max. 87 years). The most common primary tumors were mammary carcinoma (*n* = 44; 24.3%), followed by non-small cell lung carcinoma (NSCLC) (*n* = 31; 17.1%) and prostate carcinoma (*n* = 27; 14.9%). The most common pre-existing conditions were COPD (*n* = 38; 21.2%), type II diabetes (*n* = 34; 19.0%), and atherosclerosis (*n* = 25; 14.0%). Between the treatment groups (decompression/no decompression), there was no significant difference regarding age, primary tumor entity, pre-existing conditions, spinal instability score (SINS), or Karnofsky Performance Scale (KPS). See Supplementary Table S1 for details.

### 3.2. Treatment Regimes

**Figure 2:**
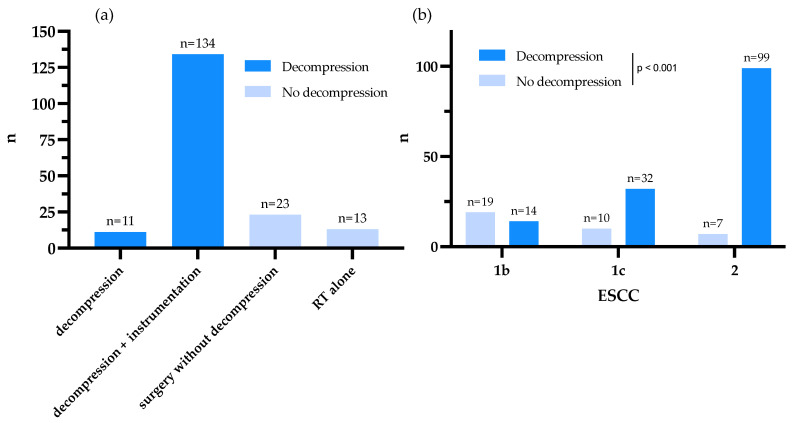
(**a**) Primary treatment approaches throughout the cohort. (**b**) ESCC distribution throughout the treatment groups. The share of patients treated with decompressive surgery increases with the ESCC.

Of the 181 treated cases, 145 (80.1%) underwent decompressive surgery, while 36 (19.9%) did not (Figure 2a). A total of 123 (82.6%) of 149 cases underwent radiotherapy. In 26 (17.4%) of the cases, radiotherapy was omitted due to the patient’s wish or a change to palliative care, and 32 cases were lost to follow-up. The share of patients receiving radiotherapy did not differ between the two treatment groups or treatment modalities (*p* = 0.541/0.057). The distribution of the ESCC grades within the two treatment groups and treatment modalities differed significantly (*p* < 0.001). While the majority of patients with ESCC 1b were treated without decompression, the percentage of patients treated with decompression increased dramatically in ESCC 1c and 2 (Figure 2b, Table 1).

### 3.3. Perioperative and Clinical Complications

The overall complication rate (including surgical and non-surgical complications) was 27.8% and did not differ between the groups (decompression vs. no decompression) or individual treatment modalities (*p* = 0.907/0.227). Similarly, there was no difference in short-term surgical complications such as wound infection (*p* = 0.689/0.458), misplaced/dislocated screws (*p* = 0.623), the need for revision surgery (*p* = 0.623/0.478), or delay of adjuvant treatment due to peri surgical complications (*p* = 0.201/0.243) (Table 2).

### 3.4. Neurological Outcome

There was no significant difference in the neurological outcome of both groups immediately after treatment (*p* = 0.469) or at last follow-up (*p* = 0.272). Three patients deteriorated to Frankel D immediately after treatment, but all three recovered to Frankel E during follow-up.

Throughout the course of the follow-up, 5 of 165 patients deteriorated to Frankel D, and 2 lost the ability to walk (Frankel C) (Table 3). One of these patients (decompression group) underwent emergent re-decompression due to local tumor recurrence and neurological worsening before starting adjuvant treatment. All other patients deteriorated due to distant metastatic disease (*n* = 5) or stroke (*n* = 1). No patient underwent emergent decompression of the treated level during their course of radiotherapy. There was no difference in the neurological outcome of patients with ESCC 1b, 1c, or 2 (Table 4).

## 4. Discussion

Next to achieving pain relief and spinal stability, the preservation or improvement of the neurological status and quality of life is one of the main goals of surgery as part of the multimodal treatment approach in patients with metastatic ESCC [10,15,16,17]. The invasiveness of surgical intervention in these patients can range from simple dorsal intraspinal decompression to complex ventrodorsal approaches such as corpectomy and instrumentation techniques in cases of impending spinal instability, in addition to ESCC. Surgery in these patients, in general, entails a high risk of perioperative complications, reported in 20% to 47% [18,19,20,21,22], and the risk for perioperative complications is likely to increase with the invasiveness of the procedure [23]. Additional intraspinal decompression in a stabilizing surgery in cases of impending spinal instability prolongs the procedure, and there is an increased blood loss associated with decompressive surgery [24]. It is common sense that in patients with a neurological deficit due to metastatic ESCC, decompressive surgery is beneficial [7]. However, for patients with low- to intermediate-grade ESCC without neurological deficit, it is far less clear whether decompression prior to RT and systemic therapy is necessary to preserve the neurological function [25]. To the best of our knowledge, this is the first study to address the question of whether additional decompressive surgery in neurologically intact patients with low- to intermediate-ESCC has an impact on the neurological outcome.

In our retrospective work, we reviewed 519 cases treated for metastatic spine disease over the course of 10 years and found that approximately one-third (34.9%) of metastatic spine patients admitted to our center presented with low- to intermediate-ESCC and no neurological deficit. While the majority of these (*n* = 145, 80.1%) underwent decompressive surgery, 36 (19.9%) were treated without decompression, which allowed for a comparison between these two groups. Regarding comorbidities that have been shown to influence outcomes [23], there was no difference among the groups. In contrast, the ESCC was significantly higher in the group treated with decompression. Due to the retrospective nature of our study, this was partly expected since, with a higher grade of ESCC, the surgeon is more likely to opt for surgical decompression [26,27]. It does, however, present a limitation to the analysis and conclusion that can be drawn from our results.

In our cohort, there was no significant difference in the number of complications, and the overall rate was comparable with previously reported studies [18,21,28]. Because the group treated without decompression included patients treated without any surgery, a comparison of intraoperative blood loss or surgery time as performed in similar studies was not reasonable [29]. While there was no statistically significant difference between the groups in any of the investigated complications, there was a larger percentage of patients in the “decompression group” in which the adjuvant treatment was delayed due to complications (11% vs. 2.9%, *p* = 0.201). The main complication responsible for delayed adjuvant treatment was wound infection (*n* = 11/17, 58.8%). Interestingly, there were no complications in the subgroup of patients treated with decompression only; however, this is likely due to the small sample size (*n* = 11) (Table 2).

Regarding the main endpoint of our study, the neurological outcome, we did not find a significant difference in either treatment group. A total of 4.5% vs. 3.2% (decompression vs. no decompression) of patients experienced deterioration of their preoperative Frankel grade throughout the course of follow-up. In only 1 of those 7 patients, neurological deterioration was due to intraspinal tumor growth at the treated level. All other patients’ deterioration was due to systemic progression of their disease or cardiovascular disease, suggesting that any of the above-mentioned treatment regimens can effectively provide adequate local disease control and should be catered to the individual patient.

Radiotherapy, in combination with tumor-specific medical options, avoids the inherent risks of surgery yet cannot address unstable metastatic disease. Using percutaneous instrumentation in unstable metastatic disease and foregoing open decompression can reduce blood loss and overall surgical time [24]. In patients with intractable pain due to spinal metastasis who are too sick to undergo extensive surgery, kyphoplasty is a valid treatment option [30,31]. Due to an increased release of circulating tumor cells after kyphoplasty, this approach should be weighed carefully in patients with oligometastatic disease [32]. Finally, in patients with limited medical options or radioresistant tumors, surgical decompression can be warranted even in neurologically asymptomatic patients.

Next to the inherent limitations of a retrospective study, our results are subject to several other limitations. The limited sample size did not allow for subgroup analysis regarding primary tumor entities or treatments, and we report on a relatively short follow-up. Most importantly, as mentioned above, there was a significant difference in ESCC severity between the two groups, making direct comparisons difficult.

There are no randomized controlled trials regarding this patient cohort, and the data on which treatment decisions are based are scarce [33,34]. Especially in the context of more effective systemic medical options for a subgroup of cancers [35], treatment approaches should always be individually tailored. Weighing whether decompressive surgery is necessary should be a conscious decision throughout that process, especially if primary tumors are likely to be radiosensitive (i.e., multiple myeloma).

## 5. Conclusions

Treatment decisions for patients without neurological deficit but low- to intermediate-ESCC (1b-2) involve several options and need to be individually tailored. In our cohort, no patient treated without decompression deteriorated due to local tumor growth throughout their follow-up, and we believe that foregoing decompressive surgery can be feasible in patients with an ESCC of 1b-2, especially in the context of radiosensitive entities (i.e., multiple myeloma, prostate carcinoma).

## Figures and Tables

**Table 1:** ESCC grade distribution and Radiotherapy within the treatment groups.

	Decompression *n* = 145		No Decompression *n* = 36		Overall *n* = 181		
	Decomp. Alone	Decomp. + Instrumentation	Surgery without Decomp	RT Alone		All Treatment Modalities *p* =	Decomp vs. No Decomp *p* =
ESCC	*n* = 11	*n* = 134	*n* = 23	*n* = 13	*n* = 181	<0.001	<0.001
1b	2 (18.2)	12 (9%)	11 (47.8%)	8 (61.5%)	33 (18.2%)		
1c	0 (0%)	32 (23.9%)	8 (34.8%)	2 (15.4%)	42 (23.2%)		
2	9 (81.8%)	90 (67.2%)	4 (17.4%)	3 (23.1%)	106 (58.6%)		
Radiotherapy	*n* = 9	*n* = 107	*n* = 20	*n* = 13	*n* = 149	0.057	0.541
Yes	7 (77.8%)	89 (83.2%)	14 (70.0%)	13 (100%)	123 (82.6%)		
No	2 (22.2%)	18 (16.8%)	6 (30.0%)	/	26 (17.4%)		

**Table 2:** Treatment-associated complications.

	Decompression *n* = 145		No Decompression *n* = 35		Overall *n* = 180		
	Decomp. Alone *n* = 11	Decomp. + Instrumentation *n* = 134	Surgery without Decomp *n* = 23	RT Alone *n* = 12		All Treatment Modalities *p*	Decomp vs. No Decomp *p*
Overall complications	0 (0%)	40 (29.9%)	7 (30.4%)	3 (25.0%)	50 (27.8%)	0.227	0.907
Wound infection	0 (0%)	9 (6.7%)	1 (4.3%)	/	10 (5.6%)	0.458	0.689
Misplaced implants	/	5 (3.7%)	2 (8.7%)	/	7 (3.9%)	0.478	0.623
Thrombosis/PE	0 (0%)	8 (6.0%)	0 (0%)	0 (0%)	8 (4.4%)	0.899	0.358
Pneumonia	0 (0%)	9 (6.7%)	1 (4.3%)	0 (0%)	10 (5.6%)	>0.999	0.689
Other *	0 (0%)	16 (11.9%)	1 (4.3%)	3 (25.0%)	20 (11.1%)	0.399	>0.999
Revision surgery	0 (0%)	16 (11.9%)	4 (17.4%)	/	20 (11.1%)	0.408	>0.999
Delay of adj. treatment due to complication	0 (0%)	16 (11.9%)	1 (2.9%)	0	17 (9.4%)	0.243	0.201

* i.e., urinary tract infection, cardiac event, delirium.

**Table 3:** Neurological outcome of treatment groups at last follow-up.

		Decompression *n* = 134	No Decompression *n* = 31	Overall *n* = 165	
Frankel Grade at last follow-up	C	1 (0.7%)	1 (3.2%)	2 (1.2%)	*p* = 0.246
	D	5 (3.7%)	0	5 (3.0%)	
	E	128 (95.5%)	30 (96.8%)	158 (95.8%)	

**Table 4:** Baseline ESCC and neurological outcome at last follow-up.

		ESCC 1b *n* = 29	ESCC 1c *n* = 39	ESCC 2 *n* = 97	Overall *n* = 165	
Frankel Grade at last follow-up	C	1 (3.4%)	0	1 (1%)	2 (1.2%)	*p* = 0.627
	D	0	1 (2.6%)	4 (4.1%)	5 (3.0%)	
	E	28 (96.6%)	38 (97.4%)	92 (94.8%)	158 (95.8%)	

## Data Availability

The datasets generated and/or analyzed in this study are available upon reasonable request.

## Supplementary Materials

The following supporting information can be downloaded at: https://www.mdpi.com/article/10.3390/cancers15020385/s1, Table S1: Clinical characteristics.
