# Supplementary material for: Role of Decompressive Surgery in Neurologically Intact Patients with Low to Intermediate Intraspinal Metastatic Tumor Burden
Source: Cancers (Basel). 2023 Jan 6;15(2):385. doi: 10.3390/cancers15020385 (PMC9857075; doi:10.3390/cancers15020385)
Supplement: Supplementary file 1 [file cancers-15-00385-s001.zip › cancers-2065459-supplementary.pdf]

Supplementary Materials: Role of Decompressive Surgery in Neurologically Intact Patients with Low to Intermediate Intraspinal Metastatic Tumor Burden

Niklas von Spreckelsen, Julian Ossmann, Maximilian Lenz, Lukas Nadjiri, Moritz Lenschow, Sergej Telentschak, Johanna Meyer, Julia Keßling, Peter Knöll, Peer Eyse, Roland Goldbrunner, Moritz Perrech, Max Scheyerer, Eren Celik, Kourosh Zarghooni and Volker Neuschmelting

Table S1. Clinical characteristics.

	Decompressive	Non-decompressive	Overall	p-Value
Number of cases	145	36	181	
Number of patients	139*	35*	173	
Number of men	80 (57.6%)	21 (60.0%)	101 (58.4%)	0.793
Number of women	59 (42.4%)	14 (40.0%)	72 (41.6%)	
Age at first consultation [years]	n = 139	n = 35	n = 173	
Mean	64.1	62.7	63.7	
SD	12.2	13.6	12.4	
Median	65	64	65	0.745
Minimum	13	32	13	
Maximum	87	84	87	
Diabetes mellitus	n = 143<br>27 (18.9%)	n = 36<br>7 (19.4%)	n = 179<br>34 (19.0%)	0.939
Atherosclerosis	n = 143<br>20 (14.0%)	n = 36<br>5 (13.9%)	n = 179<br>25 (14.0%)	0.988
COPD/Nicotine abuse	n = 143<br>29 (20.3%)	n = 36<br>9 (25.0%)	n = 179<br>38 (21.2%)	0.536
Thrombosis/PE	n = 143<br>7 (4.9%)	n = 36<br>0 (0.0%)	n = 179<br>7 (3.9%)	0.347
Obesity	n = 143<br>17 (11.9%)	n = 36<br>4 (11.1%)	n = 179<br>21 (11.7%)	>0.999
Osteoporosis	n = 142<br>4 (2.8%)	n = 36<br>3 (8.3%)	n = 178<br>7 (3.9%)	0.148
Glucocorticoid therapy	n = 143<br>10 (7.0%)	n = 36<br>4 (11.1%)	n = 179<br>14 (7.8%)	0.485
Primary tumors	n = 145	n = 36	n = 181	
Adrenal gland	1 (0.7%)	1 (2.8%)	2 (1.1%)	
Kidney	17 (11.7%)	1 (2.8%)	18 (9.9%)	
Breast	32 (22.1%)	12 (33.3%)	44 (24.3%)	
Lymphoma	5 (3.4%)	1 (2.8%)	6 (3.3%)	
SCLC	5 (3.4%)	0 (0.0%)	5 (2.8%)	
NSCLC	19 (13.1%)	12 (33.3%)	31 (17.1%)	
Thyroid gland	5 (3.4%)	1 (2.8%)	6 (3.3%)	
Multiple Myeloma	7 (4.8%)	1 (2.8%)	8 (4.4%)	0.126
Prostate	25 (17.2%)	2 (5.6%)	27 (14.9%)	
Sarcoma	4 (2.8%)	0 (0.0%)	4 (2.2%)	
CUP	7 (4.8%)	0 (0.0%)	7 (3.9%)	
Pharynx/mouth/tonsils	2 (1.4%)	1 (2.8%)	3 (1.7%)	
Gastrointestinal	10 (6.9%)	4 (11.1%)	14 (7.7%)	
Urothelium	2 (1.4%)	0 (0.0%)	2 (1.1%)	
Melanoma	2 (1.4%)	0 (0.0%)	2 (1.1%)	
Others	2 (1.4%)	0 (0.0%)	2 (1.1%)	
Cervical spine involved	n = 145	n = 36	n = 181	0.073

	25 (17.2%)	11 (30.6%)	36 (19.9%)	
Thoracic spine involved	n = 145<br>103 (71.0%)	n = 36<br>26 (72.2%)	n = 181<br>129 (71.3%)	0.888
Lumbar spine involved	n = 145<br>71 (49.0%)	n = 36<br>17 (47.2%)	n = 181<br>88 (48.6%)	0.851
Sacrum involved	n = 145<br>11 (7.6%)	n = 36<br>2 (5.6%)	n = 181<br>13 (7.2%)	>0.999
SINS	n = 144	n = 36	n= 180	
Stable (0–6)	4 (2.8%)	2 (5.6%)	6 (3.3%)	
Intermediate (7–12)	94 (65.3%)	27 (75%)	121 (67.2%)	0.228
Unstable (13–18)	46 (31.9%)	7 (19.4%)	53 (29.4%)	
KPS [%]	n = 145	n = 36	n = 181	
40	1 (0.7%)	1 (2.8%)	2 (1.1%)	
50	18 (12.4%)	3 (8.3%)	21 (11.6%)	
60	48 (33.1%)	9 (25.0%)	57 (31.5%)	0.253
70	67 (46.2%)	18 (50.0%)	85 (47.0%)	
80	11 (7.6%)	4 (11.1%)	15 (8.3%)	
90	0 (0.0%)	1 (2.8%)	1 (0.6%)	
Follow-up (months)	n = 145	n = 36	n = 181	
Median	3	9	3	
IQR	12–0	15–1	12–0.5	0.159
Minimum	0	0	0	
Maximum	79	50	79	

*The sum of patient numbers of both cohorts (n = 174) is lower than the reported cases (n = 181). If patients were treated twice but for a different metastatic lesion / spinal level 3 or more months apart. these were considered separate cases.
